# Supplementary material for: Clathrin coat controls synaptic vesicle acidification by blocking vacuolar ATPase activity
Source: eLife. 2018 Apr 13;7:e32569. doi: 10.7554/eLife.32569 (PMC5935483; doi:10.7554/eLife.32569)
Supplement: Transparent reporting form [file elife-32569-transrepform.pdf]

eLIFE

1st Floor
24 Hills Road
Cambridge CB2 1JP, UK

P 01223 855340
W elifesciences.org
T @elife

eLife's transparent reporting form

We encourage authors to provide detailed information *within their submission* to facilitate the interpretation and replication of experiments. Authors can upload supporting documentation to indicate the use of appropriate reporting guidelines for health-related research (see EQUATOR Network), life science research (see the BioSharing Information Resource), or the ARRIVE guidelines for reporting work involving animal research. Where applicable, authors should refer to any relevant reporting standards documents in this form.

If you have any questions, please consult our Journal Policies and/or contact us: editorial@elifesciences.org.

Sample-size estimation

- You should state whether an appropriate sample size was computed when the study was being designed
- You should state the statistical method of sample size computation and any required assumptions
- If no explicit power analysis was used, you should describe how you decided what sample (replicate) size (number) to use

Please outline where this information can be found within the submission (e.g., sections or figure legends), or explain why this information doesn't apply to your submission:

For comparisons between SVs and CCVs a two-sided Student's t-test for unpaired samples was used (p values indicated in the respective figures), like in Farsi et al Science 2016. In these data sets shown in Figure 2, Figure 4 and Figure 2-figure supplement 3, error bars indicate SD from more than 1000 vesicles compiled from 4-7 experimental replicates. The number of vesicles in indicated in the Figure legends, e.g. Figure 2 "...(I-J) Histograms representing the luminal pH of (I) spH-SVs (n= 3,625) and (J) spH-CCVs (n=2,233)...".

In Figure 3, error bars in (B-E) represent SD of 3-4 experimental (not technical) replicates.

In Figure 2-figure supplement 2, error bars indicate SEM of more than 500 spH-SVs. E.g., ..."(B) Cumulative curve generated from apparent luminal pH of more than 500 spH-SVs at steady state after addition of 3 mM ATP. The average luminal pH of spH-SVs after correction for bleaching was 5.82 ± 0.03 (SEM) indicating full acidification of vesicles in the presence of chloride (Miesenbock 1998)."

Replicates

- You should report how often each experiment was performed
- You should include a definition of biological versus technical replication
- The data obtained should be provided and sufficient information should be provided to indicate the number of independent biological and/or technical replicates
- If you encountered any outliers, you should describe how these were handled
- Criteria for exclusion/inclusion of data should be clearly stated
- High-throughput sequence data should be uploaded before submission, with a private link for reviewers provided (these are available from both GEO and ArrayExpress)

Please outline where this information can be found within the submission (e.g., sections or figure legends), or explain why this information doesn't apply to your submission:

We confirm that the number of independent biological repetitions have been clearly mentioned primarily in the Figure legends, and also in Results and in Methods.
Under biological replicates here we consider independent isolation of SVs or CCVs from 15+ mouse brains - this has been done at least 4, but on average 6-7 times for each experiment.
We have included all data obtained, i.e. we did not exlude any outliers.

eLife Sciences Publications, Ltd is a limited liability non-profit non-stock corporation incorporated in the State of Delaware, USA, with company number 5030732, and is registered in the UK with company number FC030576 and branch number BR015634 at the address 1st Floor, 24 Hills Road, Cambridge CB2 1JP | August 2014

Statistical reporting

- Statistical analysis methods should be described and justified
- Raw data should be presented in figures whenever informative to do so (typically when N per group is less than 10)
- For each experiment, you should identify the statistical tests used, exact values of N, definitions of center, methods of multiple test correction, and dispersion and precision measures (e.g., mean, median, SD, SEM, confidence intervals; and, for the major substantive results, a measure of effect size (e.g., Pearson's r, Cohen's d)
- Report exact p-values wherever possible alongside the summary statistics and 95% confidence intervals. These should be reported for all key questions and not only when the p-value is less than 0.05.

Please outline where this information can be found within the submission (e.g., sections or figure legends), or explain why this information doesn't apply to your submission:

For each experiment, we have identified the statistical tests used, p values, and error. These information have been mentioned primarily in the Figure legends, but also in Results and in Methods.

(For large datasets, or papers with a very large number of statistical tests, you may upload a single table file with tests, Ns, etc., with reference to sections in the manuscript.)

Group allocation

- Indicate how samples were allocated into experimental groups (in the case of clinical studies, please specify allocation to treatment method); if randomization was used, please also state if restricted randomization was applied
- Indicate if masking was used during group allocation, data collection and/or data analysis

Please outline where this information can be found within the submission (e.g., sections or figure legends), or explain why this information doesn't apply to your submission:

- sample allocation into experimental group - not applicable
- sample masking was done at the level of analysis: here, one scientist has performed data collection, the other scientist has analyzed data

Additional data files ("source data")

- We encourage you to upload relevant additional data files, such as numerical data that are represented as a graph in a figure, or as a summary table
- Where provided, these should be in the most useful format, and they can be uploaded as "Source data" files linked to a main figure or table
- Include model definition files including the full list of parameters used
- Include code used for data analysis (e.g., R, MatLab)
- Avoid stating that data files are "available upon request"

Please indicate the figures or tables for which source data files have been provided:

Figure 1 and Figure supplement 2: The reconstructed CCV particle (with the vesicle inside) is deposited with the EMDB-ID #4335.
Table 1: The mass spectometry data table contains original readouts as obtained from the MS facility.

The custom script cited here is a part of larger algorithm that is written by Burkhard Rammner for the Sciloop company, and it is a property of the Sciloop. The script enables and simplifies the work with the published structures, and it is not essential for any data point presented here. We are extracting the key aspects of the script now, and will make it publicly available through the GitHub repository when the ms is accepted for publication.
